# Supplementary material for: Cryo-EM structures of remodeler-nucleosome intermediates suggest allosteric control through the nucleosome
Source: eLife. 2019 Jun 18;8:e46057. doi: 10.7554/eLife.46057 (PMC6611695; doi:10.7554/eLife.46057)
Supplement: Supplementary file 1. [file elife-46057-supp1.docx]

| **Key Resources Table** | | | | |
| --- | --- | --- | --- | --- |
| **Reagent type (species) or resource** | **Designation** | **Source or reference** | **Identifiers** | **Additional information** |
| strain, strain background (*Escherichia coli*) | Rosetta (DE3) | Millipore sigma | 70954 | Chemically competent cells |
| strain, strain background (*Escherichia coli*) | BL1 (DE3) pLysS | Agilent Technologies | 200132 | Chemically competent cells |
| recombinant DNA reagent | core Widom 601  (bold) and  flanking DNA  sequences | (Lowary and Widom, 1998) |  | 5’-CGGCCGCC**CTGGAGAATCCCGGTGCCGAGGCCGCTCAATTGGTCGTAGACAGCTCTAGCACCGCTTAAACGCACGTACGCGCTGTCCCCCGCGTTTTAACCGCCAAGGGGATTACTCCCTAGTCTCCAGGCACGTGTCAGATATATACATCCTGT**GCATGTATTGAACAGCGACCTTGCCGGTGCCAGTCGGATAGTGTTCCGAGCTCCCACTCTAGAGGATCCCCGGGTACC-3’ |
| recombinant DNA reagent | 601 plasmid | (Lowary and Widom, 1998) |  | PCR template |
| recombinant DNA reagent | pBH4-SNF2h | (Leonard and Narlikar, 2015) |  | Expression plasmid |
| recombinant DNA reagent | Pet3a-H2A | (Yang et al., 2006) |  | Expression plasmid |
| recombinant DNA reagent | Pet3a-H2B | (Yang et al., 2006) |  | Expression plasmid |
| recombinant DNA reagent | Pet3a-H3 | (Yang et al., 2006) |  | Expression plasmid |
| recombinant DNA reagent | Pet3a-H4 | (Yang et al., 2006) |  | Expression plasmid |
| sequence-based reagent | 601 core forward primer | IDT |  | 5’-CTGGAGAATCCCGGTGCCG-3’ |
| sequence-based reagent | 601 +60 reverse primer | IDT |  | 5’-AGAGTGGGAGCTCGG AACAC-3’ |
| sequence-based reagent | Cy3- 601 core forward primer | IDT |  | 5’-/Cy3/ CTGGAGAATCCCGGTGCCG-3’ |
| sequence-based reagent | Cy5- 601 -9 forward primer | TriLink Biotechnologies |  | 5’-{Cyanine5-C6-NH}GCGGCC GCCCTGGAGAATCC-3’ |
| sequence-based reagent | Bio- 601 +78 reverse primer | IDT |  | 5’-/5BioTeg/GGTACCCGG GGA TCCTCTAGAG-3’ |
| sequence-based reagent | 601 -120F C149cy5 | Iba |  | 5’-GGCACGTGTCAGATATATACATCCTGTG5ATGTATTGAACA-3’  5= cy5-C6-Amino-2'deoxycytidine |
| sequence-based reagent | SNF2h K298E forward primer | IDT |  | 5’-GAGAAGTCTGTGTTCGAAAAATTTAATTGGAG-3’ |
| sequence-based reagent | SNF2h K298E reverse primer | IDT |  | 5’-CTCCAATTAAATTTTTCGAAC ACAGACTTCTC-3’ |
| sequence-based reagent | SNF2h K440A forward primer | IDT |  | 5’-CTCAACTCAGCAGGCGCGATGGACAAAATGAGG-3' |
| sequence-based reagent | SNF2h K440A reverse primer | IDT |  | 5’-CCTCATTTTGTCCATCGCGCCTGCTGAGTTGAG-3' |
| sequence-based reagent | SNF2h D442A forward primer | IDT |  | 5’-CTCAGCAGGCAAGATGGCGAAAATGAGGTTATTGAAC-3' |
| sequence-based reagent | SNF2h D442A reverse primer | IDT |  | 5’-GTTCAATAACCTCATTTTCGCCATCTTGCCTGCTGAG-3' |
| sequence-based reagent | SNF2h K443A forward primer | IDT |  | 5’-CAGCAGGCAAGATGGACGCGATGAGGTTATTGAACATC-3' |
| sequence-based reagent | SNF2h K443A reverse primer | IDT |  | 5’-GATGTTCAATAACCTCATCGCGTCCATCTTGCCTGCTG-3' |
| sequence-based reagent | SNF2h N448A forward primer | IDT |  | 5’-GACAAAATGAGGTTATTGGCGATCCTAATGCAGTTGAG-3' |
| sequence-based reagent | SNF2h N448A Reverse primer | IDT |  | 5’-CTCAACTGCATTAGGATCGCCAATAACCTCATTTTGTC-3' |
| sequence-based reagent | SNF2h W581A forward primer | IDT |  | 5’-GTAATTTTGTATGATTCTGATGCGAATCCCCAAGTAGATCTTC-3' |
| sequence-based reagent | SNF2h W581A reverse primer | IDT |  | 5’-GAAGATCTACTTGGGGATTCGCATCAGAATCATACAAAATTAC-3' |
| peptide, recombinant protein  (*Homo sapiens*) | SNF2h | (Leonard and Narlikar, 2015) |  |  |
| peptide, recombinant protein  (*Xenopus laevis*) | Histone H2A | (Luger et al., 1997) |  |  |
| peptide, recombinant protein  (*Xenopus laevis*) | Histone H2B | (Luger et al., 1997) |  |  |
| peptide, recombinant protein  (*Xenopus laevis*) | Histone H3 | (Luger et al., 1997) |  |  |
| peptide, recombinant protein  (*Xenopus laevis*) | Histone H4 | (Luger et al., 1997) |  |  |
| peptide, recombinant protein  (*Xenopus laevis*) | Histone H3 33C | (Rowe and Narlikar, 2010) |  |  |
| peptide, recombinant protein  (*Escherichia virus T4*) | T4 DNA Ligase | New England Biolabs | Cat #: M0202L |  |
| peptide, recombinant protein  (*Bos taurus*) | Catalase | Sigma | Cat #: E3289 |  |
| peptide, recombinant protein  (*Aspergillus niger*) | Glucose oxidase | Sigma | Cat #: G2133 |  |
| peptide, recombinant protein  (*Oryctolagus cuniculus*) | Lactate dehydrogenase | Sigma | Cat #: 427217 |  |
| peptide, recombinant protein  (*Oryctolagus cuniculus* ) | Pyruvate Kinase | Sigma | Cat #: 10128155001 |  |
| chemical compound, drug | ATP | GE | Cat #: 27-2056-01 |  |
| chemical compound, drug | γ-^32^P-ATP | Perkin Elmer | Cat #: Blu002Z250uC |  |
| chemical compound, drug | ADP | Millipore sigma | Cat #: 117105 |  |
| chemical compound, drug | Cy3-maleimide | Lumiprobe | Cat #: 21080 |  |
| chemical compound, drug | Cy5-maleimide | Lumiprobe | Cat #: 43080 |  |
| chemical compound, drug | dNTPs | Allstar Scientific | Cat #: 471-5DN |  |
| chemical compound, drug | N-(2-aminoethyl)-3-aminopropyltrimethoxysilane | United Chemicals | Cat #: A0700 |  |
| chemical compound, drug | mPEG-SVA | Laysan Bio |  |  |
| chemical compound, drug | biotin-PEG-SVA | Laysan Bio |  |  |
| chemical compound, drug | acetylated BSA | Promega | Cat #: R3691 |  |
| chemical compound, drug | Neutravidin | Life Technologies | A2666 |  |
| chemical compound, drug | Trolox | Sigma | Cat #: 238813 |  |
| chemical compound, drug | 10XTBE | Bio-Rad | Cat #: 161-0770 |  |
| chemical compound, drug | Acrylamide/Bis-acrylamide | Bio-rad | Cat #: 161-0146 |  |
| chemical compound, drug | HEPES | Fisher | Cat #: BP310 |  |
| chemical compound, drug | Tris Base | Thermo Fisher | Cat #: BP1525 |  |
| chemical compound, drug | NaCl | RPI | Cat #: S23020 |  |
| chemical compound, drug | KCl | Sigma | Cat #: P3911 |  |
| chemical compound, drug | MgCl_2_ | RPI | Cat #: M24000 |  |
| chemical compound, drug | Glycerol | Sigma | Cat #: G7893 |  |
| chemical compound, drug | NP40 (IGEPAL) | Sigma | Cat #: I8896 |  |
| chemical compound, drug | 2-Mercaptoethanol | Sigma | Cat #: M3148 |  |
| chemical compound, drug | Glucose | RPI | Cat#: G32045 |  |
| chemical compound, drug | NADH | Millipore Sigma | Cat#: 481913 |  |
| chemical compound, drug | Phosphoenol Pyruvate | Thermo Fisher | Cat#: NC9842221 |  |
| software, algorithm | Prism 6 | Graphpad |  |  |
| software, algorithm | Traces | [https://github.com/stephlj/Traces](https://github.com/stephlj/Traces) |  |  |
| software, algorithm | pyhsmm | [https://github.com/mattjj/pyhsmm](https://github.com/mattjj/pyhsmm) |  |  |
| software, algorithm | Slopey | [https://github.com/stephlj/slopey](https://github.com/stephlj/slopey) |  |  |
| software, algorithm | PyEM | <https://github.com/asarnow/pyem> |  |  |
| software, algorithm | Gautomatch | <http://www.mrc-lmb.cam.ac.uk/kzhang/> |  |  |
| software, algorithm | SerialEM | (Mastronarde, 2005) |  |  |
| software, algorithm | RELION 3.0 | {Zivanov:2018es} |  |  |
| software, algorithm | Motioncor2 | (Zheng et al., 2017) |  |  |
| software, algorithm | GCTF | (Zhang, 2016) |  |  |
| software, algorithm | UCSFImage4 | (X. Li et al., 2015) |  |  |
| software, algorithm | EMAN2 | (Tang et al., 2007) |  |  |
| software, algorithm | CryoSPARC | (Punjani et al., 2017) |  |  |
| software, algorithm | Diffmap.exe | http://grigoriefflab.janelia.org/diffmap |  |  |
| software, algorithm | Coot | (Emsley et al., 2010) |  |  |
| software, algorithm | Phenix | (Adams et al., 2010) |  |  |
| software, algorithm | ImageJ | <https://imagej.nih.gov/ij/> |  |  |
| other | Superdex 200 increase 10/300 GL | GE | Cat. #: 29091596 |  |
| other | HiTrap QXL column | GE | Cat. #: 17-5159-01 |  |
| other | Superdex 200 HiLoad 26/600 | GE | Cat. #: 28989336 |  |
| other | TALON metal affinity resin | Clontech | Cat. # 635503 |  |
